# Net-Winged Midge Genus *Blepharicera* Macquart (Diptera: Blephariceridae) in China: The First DNA Barcode Database with Descriptions of Four New Species and Notes on Distribution

**DOI:** 10.3390/insects13090794

**Published:** 2022-08-31

**Authors:** Xiao Zhang, Ding Yang, Zehui Kang

**Affiliations:** 1 Shandong Engineering Research Center for Environment-Friendly Agricultural Pest Management, College of Plant Health and Medicine, Qingdao Agricultural University, Qingdao 266109, China; 2 College of Plant Protection, China Agricultural University, Beijing 100193, China

**Keywords:** aquatic insects, DNA barcoding, taxonomy, Chinese fauna, identification key, COI

## Abstract

**Simple Summary:**

Blephariceridae is a small but highly specialized family in Diptera with extreme morphological adaptations for living in fast-moving waters. Among the seven blepharicerid genera in China, *Blepharicera* Macquart, 1843 is the species richest genus with 12 known species. There is a serious gap in knowledge concerning larval stages and connecting them to adult morphology. At present, DNA barcoding using mitochondrial (mt) cytochrome c oxidase subunit 1 gene (COI) has proven to be successful for identification in many animal groups. There are about 60 species of the genus *Blepharicera* known in the world. However, only one identified *Blepharicera* species from Europe is currently available in DNA sequence repositories, and a DNA barcode database is still lacking for Chinese *Blepharicera*. To fill this gap, we started a project with the objectives to collect specimens of *Blepharicera* across China, identify the species by morphological characters, and build an mt COI barcode database for this important insect group. Molecular and morphological markers show that four morphospecies examined represent members of the genus *Blepharicera* and constitute four new, distinct species. This initial attempt indicates the validity of mt COI barcodes for *Blepharicera* in delimiting species and contributes to the growing library of DNA barcodes of net-winged midges of the world.

**Abstract:**

Mitochondrial (mt) cytochrome c oxidase 1 (COI) gene is more and more widely used for DNA barcoding, which provides a rapid and timely identification as this technique is not limited by polymorphism, sex, and life stages and fundamentally complements traditional evolutionary taxonomy. The present study generated 33 mt COI sequences of seven Chinese *Blepharicera* Macquart, 1843 species with an average of 594 bp, which represent the first DNA barcode database for Chinese *Blepharicera*. Genetic distance analysis reveals that intraspecific distances in the genus are generally less than 1.7%, and interspecific distances range from 5.4% to 20.3%. Phylogenetic analysis shows that each species recovered in our analyses is separated from all neighboring species. Based on molecular and morphological data, four *Blepharicera* species from China, *B. beishanica* **sp. nov.**, *B. dushanzica* **sp. nov.**, *B. nigra* **sp. nov.** and *B. xinjiangica* **sp. nov.**, are described and illustrated as new to science. Identification keys for adults and larvae of Chinese *Blepharicera* are also presented. Geographical analysis shows that Southwest China is the species’ richest region. Our results will be useful in tackling taxonomic problems, understanding species distribution, and resolving nomenclature conflicts associated with *Blepharicera* species.

## 1. Introduction

Members of the family Blephariceridae, also known as net-winged midges, are a small but highly specialized group in Diptera. Their larvae and pupae have extreme morphological adaptations for living on smooth rocks and boulders in fast-moving waters, including ventral adhesive discs and a compact cephalic division [1]. Based on these morphological characteristics generally associated with larval habitat preference for fast-flowing streams, Blephariceridae is considered to form the infraorder Blepharicerimorpha together with the families Deuterophlebiidae and Nymphomyiidae [2,3,4,5,6], but current molecular data support that Blephariceridae is nested in infraorder Psychodomorpha [7,8,9]. Adult net-winged midges are often found close to the natal stream resting on the underside of vegetation or logs [10]. Because their habitats contain clear and well-oxygenated mountain streams, some blepharicerid species are known to be sensitive bioindicators [11,12]. Females of many species are insect predators, sucking the blood of soft-bodied aquatic insects (e.g., mayflies and stoneflies) and slow-flying Diptera (e.g., Chironomidae, Dixidae, Tipulidae, and even other smaller Blephariceridae) [10], while females of some species are nectarivores [1].

Net-winged midges are distributed on most major continents and several continental islands with about 330 extant species. These species are grouped into 30 genera, of which *Blepharicera* Macquart, 1843 is one of the most common and largest genera with about 60 species [1]. The genus *Blephariceria* can be easily distinguished from other genera of Blephariceridae by the following features: head normally dichoptic in male and subholoptic in female; antennae with 13 flagellomeres; mandible absent in male and often present in female; middle coxa of a female with setose median outgrowth; the base of hind basitarsus with obvious black setae; claws nonsetate dorsally; vein R with 3 branches, veins R_4_ and R_5_ separate for entire length; absence of cross vein bm-cu, presence of M_2_ [10,13,14]. In *Blephariceria*, eyes, and mouthparts are usually sexually dimorphic.

The genus *Blepharicera* has the most species and the widest distribution among the seven blepharicerid genera in China. At present, 12 *Blephariceria* species have been recorded from China, of which five species were described by different entomologists between 1937 and 1990 [13,15,16,17], while the remaining seven species (six new species and one new record) were published by Kang et al. [18,19,20]. However, larvae of only three Chinese *Blepharicera* species are known, including *B. uenoi* (Kitakami, 1937), which was published only based on larvae.

At present, DNA barcoding using a fragment of the mitochondrial (mt) cytochrome c oxidase subunit 1 gene (COI) has proven to be successful for identification in many dipteran groups, which provides a rapid and timely identification as this method is not limited by polymorphism, sex, and life stages and fundamentally complements traditional evolutionary taxonomy [21,22,23]. DNA Barcoding usually has considerable congruence with morphology-based identifications [24,25,26]. Furthermore, it has revealed cryptic diversity within morphospecies of some insect taxa [27,28] or indicated the presence of one species despite morphological variation within studied specimens [29].

Despite the wide use of mt COI barcoding in current taxonomy and biodiversity studies, the method has been only rarely used in taxonomic studies of *Blepharicera*. Only one identified *Blepharicera* species from Europe is currently available in DNA sequence repositories, and a DNA barcode database is still lacking for the Chinese fauna of *Blepharicera*. To fill this gap, we started a project with the objectives to collect specimens of *Blepharicera* across China, identify the species by morphological characters and build a DNA barcode database for this important insect group. In this study, the first DNA barcode database for Chinese *Blepharicera* with 33 mt COI sequences from seven species is presented. Based on molecular and morphological data, four *Blepharicera* species from China are described and illustrated as new to science. Identification keys for adults and larvae of Chinese *Blepharicera* and notes on the distribution of the genus in China are also provided.

## 2. Materials and Methods

### 2.1. Specimen Preparation, Observation, and Description

Specimens for this study were collected from several localities in China by insect net or light trap. Studies were based on whole-animal preparations and dissections. Photographs were captured by a Canon EOS 90D digital camera through a macro lens. Preparations of male genitalia were made by immersing the apical portion of the abdomen in warm lactic acid for 0.5–1.0 h. Specimens were examined and illustrations were prepared by using a ZEISS Stemi 2000-C stereomicroscope. After examination, the removed abdomen was transferred to fresh glycerine and stored pinned in a microvial. Type specimens of the new species in this study were deposited in the Entomological Museum of China Agricultural University, Beijing, China (CAU), and the Entomological Museum of Qingdao Agricultural University, Shandong, China (QAU). Structural terminology is based on Courtney [30] and Jacobson et al. [14].

### 2.2. DNA Extraction, Amplification, and Sequencing

Thirty-three specimens of seven Chinese *Blepharicera* species were sequenced in this study (Table 1). Specimens for DNA extraction were preserved in absolute ethanol at −20 °C for long-term storage at Qingdao Agricultural University, Qingdao, China (QAU). The DNA was extracted from muscle tissue of the thorax or leg using the DNeasy Blood and Tissue kit (Qiagen) according to the manufacturer’s protocol. The DNA barcode region (mt COI gene) was amplified and sequenced from all specimens using universal primers LCO1490: 5′-GGGTCAACAAATCATAAAGATATTGG-3′ and HCO2198: 5′-TAAACTTCAGGGTGACCAAAAAATCA-3′ [31]. All PCR reactions were performed in a 25 μL volume containing 12.5 μL Taq PCR Master Mix, 1.0 μL of DNA extract, 1.0 µL primer LCO1490, 1.0 µL Primer HCO2198, 9.5 µL ddH_2_O. The cycling profile was 94 °C for 4 min, 30 cycles of 94 °C for 30 sec, 45 °C for 30 sec, 72 °C for 1 min, and a final extension period of 72 °C for 10 min. Successful PCR products were purified and sequenced by Sangon Biotech (Shanghai, China).

### 2.3. Phylogenetic Analysis

A total of 39 mt COI sequences of 11 species were used for phylogenetic analysis, of which 33 sequences of seven Chinese *Blepharicera* species are newly sequenced in this study, and three sequences of *B. fasciata* (Westwood, 1842) from Europe were downloaded from Barcode of Life Data Systems (BOLD) [32] with the sequence IDs RODI013-20, RODI014-20 and RODI015-20, and three sequences of three *Liponeura* Loew, 1844 (Diptera: Blephariceridae) species as outgroup were downloaded from GenBank of NCBI [33] with the accession nos. KX453756, MH407232 and MH407233. Mitochondrial COI sequences were aligned by codons using MUSCLE implemented in MEGA7 [34]. The aligned sequences were then analyzed using MEGA7 to generate Neighbor-Joining (NJ) and Maximum Likelihood (ML) trees [34] and using MrBayes v3.2.2 to generate Bayesian inference (BI) tree [35]. The NJ and ML trees were conducted with the Kimura 2-parameter model and the reliability of the inferred topology was assessed by performing 1000 rapid bootstrap replicates. The BI tree was conducted with the GTR+I+G model and two simultaneous runs of one million generations.

## 3. Results

### 3.1. Molecular Analysis

The present study generated 33 mt COI sequences of seven Chinese *Blepharicera* species with an average of 594 bp, which represent the first DNA barcode database for Chinese *Blepharicera*. Three mt COI sequences of the European species *B. fasciata* downloaded from BOLD were also used for analysis. The 36 studied mt COI sequences are found to belong to eight species, of which the following four are described as new species: *B. beishanica* **sp. nov.**, *B. dushanzica* **sp. nov.**, *B. nigra* **sp. nov.** and *B. xinjiangica*.

**sp. nov**. Genetic distances of the sequences conducted using the Kimura 2-parameter model in MEGA7 [34] and are shown in Table 2 and Table S1. Intraspecific distances of the sequences in *Blepharicera* are generally less than 1.7%, and the maximum intraspecific distance exists in *B. nigra* **sp. nov.** from China. Interspecific distances range from 5.4% (between *B. dushanzica* **sp. nov.** and *B. xinjiangica* **sp. nov.**) to 20.3% (between *B. beishanica* **sp. nov.** and *B. kongsica* Zhang *et* Kang, 2022).

**Figure 1:**
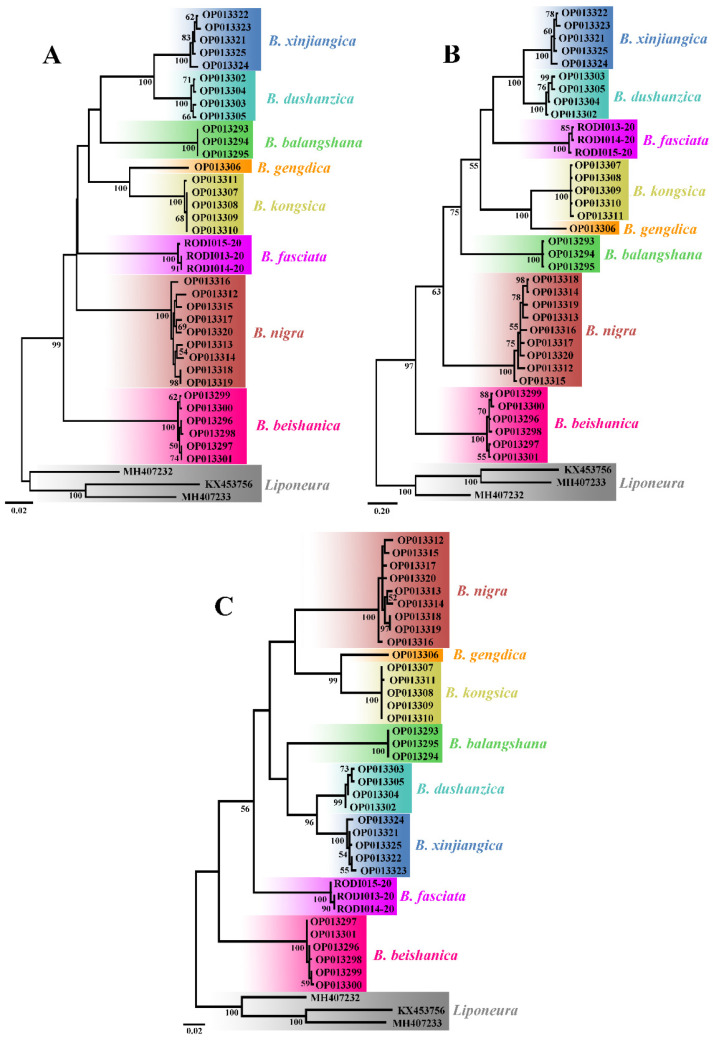
Neighbor-Joining (**A**), Bayesian inference (**B**), and Maximum Likelihood (**C**) trees demonstrating the clustering of *Blepharicera* Macquart, 1843 COI sequences. Bootstrap values and posterior probabilities of 50 and above are indicated.

All the 36 COI sequences of the eight *Blepharicera* species as ingroup and three mt COI sequences of three *Liponeura* species as outgroup are used to construct NJ, BI, and ML trees. Each tree shows eight distinct clades, as shown in Figure 1, which means that each species recovered in our analyses is separated from all neighboring species.

### 3.2. Taxonomy

The genus *Blepharicera* has the most species among the seven blepharicerid genera in China with 12 known species. Examination of specimens from several localities in China revealed four new *Blepharicera* species: *B. beishanica* **sp. nov.** from Qinghai, *B. dushanzica* **sp. nov.** from Xinjiang, *B. nigra* **sp. nov.** from Yunnan, and *B. xinjiangica* **sp. nov.** from Xinjiang. Based on the type and non-type specimens and literature, we present a dichotomous key for adult males of all Chinese *Blepharicera* species except for *B. uenoi*, whose adults are unknown. The larvae of only three Chinese species including *B. uenoi* are known, and a key for them is also presented here.

#### 3.2.1. *Blepharicera* *beishanica* sp. nov.

urn:lsid:zoobank.org:act:2D8E7B7C-ED6B-45F9-B4DB-5D8CE81273BE

**Type material. *Holotype*:** male (CAU), China: Qinghai Province, Huzhu County, Beishan Forest Farm (36°50’56’’ N 101°56’47’’ E, 2100 m), 2015.VII.27, Liang Wang; ***Paratypes*:** 10 males & 3 females (QAU), same data as holotype; 1 female (QAU), China: Qinghai Province, Huzhu County, Beishan Forest Farm (36°50′56″ N 101°56′47″ E, 2100 m), 2015.VII.26, Liang Wang (light trap).

**Diagnosis.** Compound eye with dorsal division 1/15 of ventral division in male. Mesonotum brown with a yellow median stripe at the posterior half. Mesopleuron is mostly light brown except for anepimeron light yellow. Rs 1.5 times as long as r-m. Gonostylus is slightly swollen and notched apically. Dorsal paramere with tip arrowhead-shaped; dorsal carina clearly visible, tip round and blunt. Medial depression of 8th sternite flat in female. Genital fork X-shaped in female.

**Description. Male.** Body length 4.00–5.00 mm; wing length 5.25–6.00 mm, width 2.00–2.25 mm.

**Figure 2:**
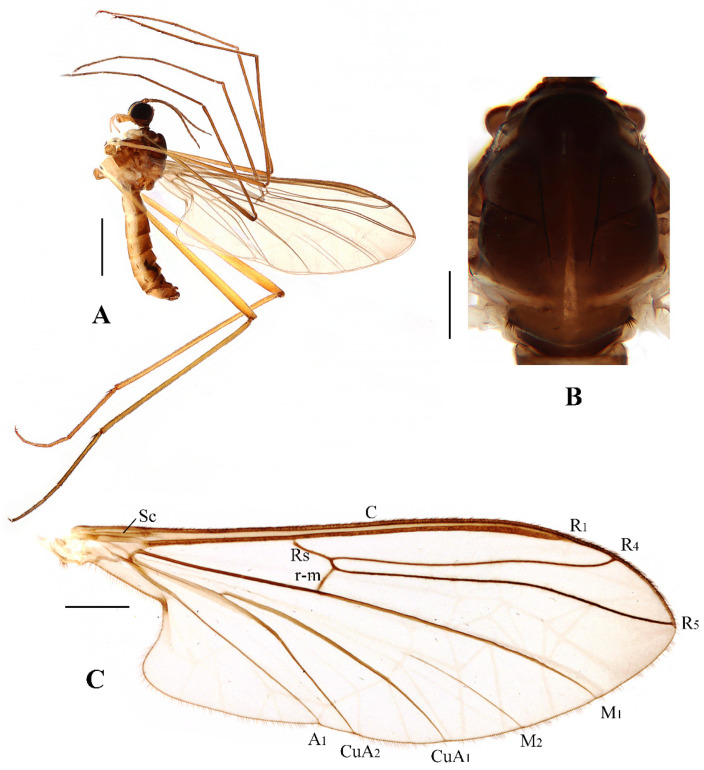
*Blepharicera beishanica***sp. nov.** (male holotype). (**A**) habitus of male, lateral view. (**B**) thorax, dorsal view. (**C**) wing. Scale bars: 1.0 mm (**A**), 0.25 mm (**B**), 0.5 mm (**C**).

**Figure 3:**
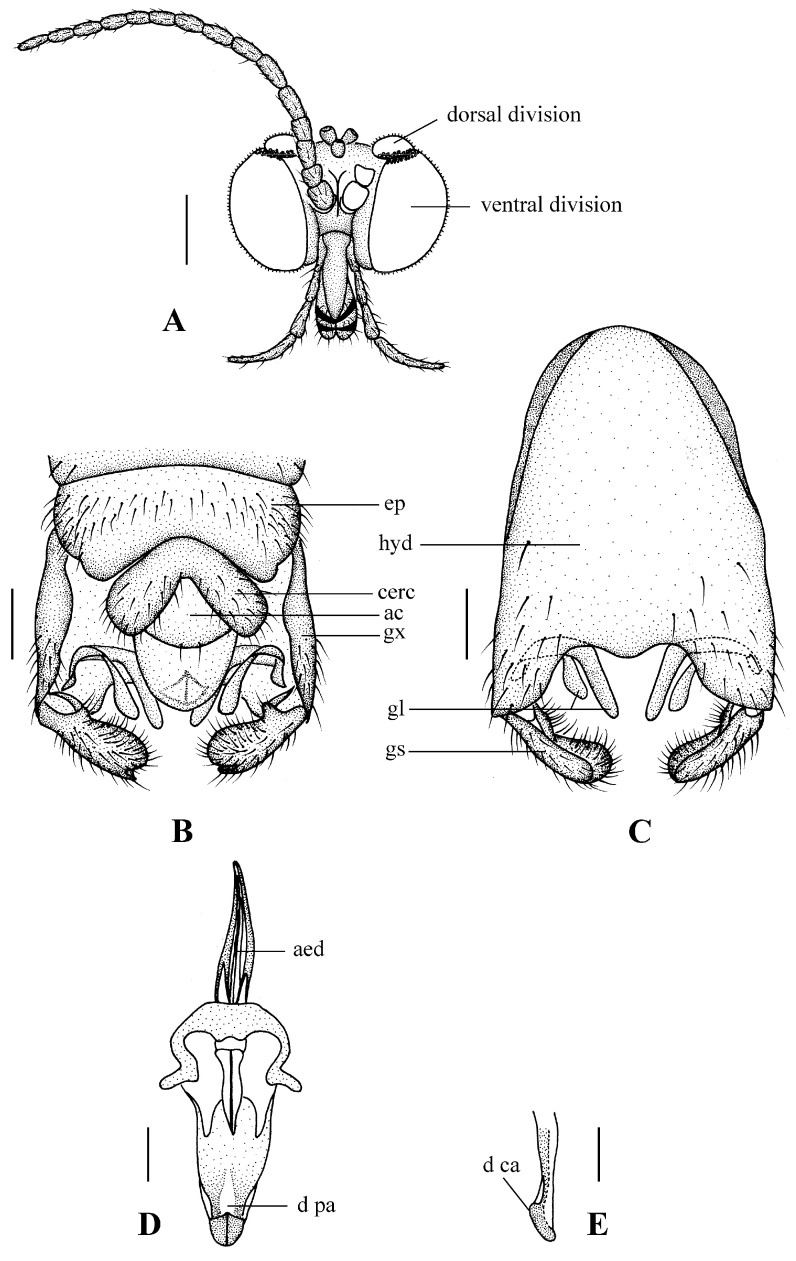
*Blepharicera beishanica***sp. nov.** (male holotype). (**A**) male head, frontal view (**B**) male genitalia, dorsal view (**C**) male genitalia, ventral view (**D**) aedegal complex, dorsal view (**E**) tip of dorsal paramere, lateral view. Scale bars: 0.25 mm (**A**), 0.1 mm (**B**–**E**). Abbreviations: ac = anal cone, aed= aedeagus, cerc = cercus, d ca = dorsal carina, d pa = dorsal paramere, ep = epandrium, gl = gonocoxal lobe, gs = gonostylus, gx = gonocoxite, hyd = hypandrium.

Head (Figure 2A and Figure 3A) uniformly brown with brownish black hairs. Compound eyes dichoptic, interocular ridge absent; each compound eye divided, callis oculi absent; dorsal division contiguous with ventral division, 1/15 of ventral division; dorsal division with 11–12 rows of ommatidia, ommatidia red-orange, larger in diameter, with ommatrichia; ventral division black with ommatrichia. Ocelli brownish yellow. Scape and pedicel oval, brown with brownish black hairs; first flagellomere constricted at base, widening at apex, basal 1/2 brownish yellow, apical 1/2 brown, with brownish-black hairs; other flagellomeres cylindrical, brown with brownish black hairs; ultimate flagellomere 1.3 times length of penultimate flagellomere. Clypeus nearly rectangular, brownish yellow, as long as wide; labrum yellow; labellum yellow with brownish black hairs; proboscis about 0.47 times the length of head width. Palpus with five segments, 1st segment almost invisible; 2nd and 3rd segments cylindrical, yellow with brown hairs; 4th segment cylindrical, slightly swollen apically, yellow with brown hairs; 5th segment slender, brownish yellow with brown hairs; relative length of distal four segments as 1.0:1.0:1.5:3.4.

Thorax (Figure 2B). Pronotum and propleuron brown without hairs. Mesonotum brown with a yellow median stripe at posterior 1/2; scutellum light brown with numerous hairs grouped at the posterolateral corner; metanotum brown; mesopleuron mostly light brown except anepimeron light yellow. The relative length of the femur, tibiae, and 1st to 5th tarsomeres in the fore leg as 11.7:10.3:6:2.8:1.7:0.8:1, in the mid-leg as 13.3:10.8:5.2:2.7:1.5:0.8:1, in the hind leg as 17:15:5.5:1.5:1:0.8:1. Fore coxa light brown with brown hairs; mid coxa with an appendage, pale with brownish black hairs; hind coxa pale with brownish black hairs; trochanters pale, anterior margin with black spot apically, with brownish-black hairs; fore and mid femora brownish yellow basally and gradually darkened to brown apically, with brownish-black hairs; hind femur brownish yellow basally and gradually darkened to light brown apically, with brownish-black hairs; fore and mid tibiae brown with brownish black hairs; hind tibia light brown with brownish black hairs; tarsomeres brown with brownish black hairs; claw brown. Tibial spurs 0–0–0. Wing (Figure 2C) slightly brown apically, apical 1/3 of sc brown; veins brown. Sc rudimentary, not ending at the base of Rs; Rs slightly curved basally, 1.5 times as long as r-m; R_4_ wavy, the length from the end of R_1_ to end of R_4_ shorter than the length from the end of R_4_ to end of R_5_; r-m straight, included angle between r-m and Rs less than 90 degrees; the length from the end of M_1_ to end of M_2_ as long as the length from the end of M_2_ to end of CuA_1_. The base of the halter is pale, and the apex of the halter is light brown with brownish-black hairs.

Abdomen. First tergum brown with middle area pale, 2nd to 7th terga light brown with brown strips, 8th tergum brownish yellow; 1st sternum light brown, 2nd to 7th sterna light brown with pale stripes laterally; abdomen with brownish black hairs. Male genitalia (Figure 3B–E) brown with basal 1/2 hypandrium pale. Epandrium trapeziform, posterior margin concave, with several brown hairs. Cercus triangular, inner margin slightly bulge, with several brown hairs; anal cone round with two long hairs apically. Gonostylus slightly swollen and notched apically, outer side with a wide triangular lobe folded ventrally, with hairs. Gonocoxal lobe bifurcated, outer gonocoxal lobe transparent, rod-shaped, curved medially swollen apically; inner gonocoxal lobe transparent, rod-shaped, nearly straight. Hypandrium rectangular, twice as long as wide, slightly narrow basally, posterior margin concave, with several brown hairs. Aedeagus slender, curved backward; dorsal paramere with tip arrowhead-shaped and posterior margin round; dorsal carina clearly visible, tip round and blunt.

**Female**. Body length 5.50–6.50 mm, wing length 7.25–8.00 mm, wing width 2.50–2.80 mm.

**Figure 4:**
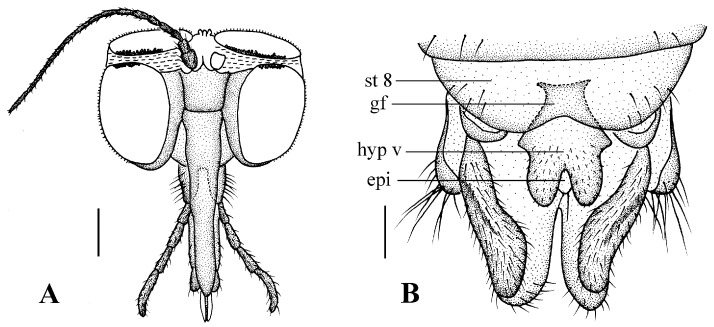
*Blepharicera beishanica***sp. nov.** (female paratype). (**A**) female head, frontal view (**B**) female terminalia, ventral view. Scale bars: 0.25 mm (**A**), 0.1 mm (**B**). Abbreviations: epi = epiproct, gf = genital fork, hyp v = hypogynial valve, st 8 = eight sternite.

Head (Figure 4A). Compound eyes subholoptic, interocular ridge present; each compound eye divided, callis oculi present; dorsal division separated from ventral division, as large as ventral division; dorsal division with about 20 rows of ommatidia, ommatidia red-orange, larger in diameter, with ommatrichia; ventral division black with ommatrichia. Scape and pedicel oval, dark brown with brownish black hairs; first flagellomere constricted at base, widening at apex, basal 1/2 brownish yellow, apical 1/2 dark brown, with brownish-black hairs; other flagellomeres cylindrical, tapering apically, dark brown with brownish black hairs; ultimate flagellomere twice length of penultimate flagellomere. Labrum brown; labellum brownish yellow with brownish black hairs; mandible brownish yellow; proboscis about 0.89 times length of head width. Palpus with five segments, 1st segment almost invisible, brownish yellow with brownish black hairs; 2nd to 4th segments cylindrical, brownish yellow with brownish black hairs; 5th segment slender, cylindrical, brownish yellow with apical 1/2 brownish black, with brownish-black hairs; relative length of distal four segments as 1.0:1.5:1.5:2.5. Tibial spurs 0–0–2. Terminalia (Figure 4B): 8th sternite approximately trapezoidal, bilobate posteriorly, medial depression flat, with several hairs laterally; genital fork X-shaped; hypogynial plate broad basally, with triangular bulge laterally, bilobate posteriorly, each lobe round apically, intervalvular area V-shaped, with short hairs posteriorly; epiproct conical with two prominent hairs apically; spermathecae three in number.

**Distribution.** Currently known only from China (Qinghai).

**Etymology.** The specific name refers to the type locality Beishan Forest Farm.

**Remarks.** This new species is very similar to *B. indica* (Brunetti, 1911) from Afghanistan, Pakistan, Sri Lanka, and India but can be separated by the dorsal division being contiguous with ventral division and being about 1/15 of ventral division, and the gonostylus being broad and distinctly notched apically. In *B. indica*, the dorsal division is separated from ventral division by a distinct suture and is as large as ventral division, and the gonostylus is distinctly slender and not distinctly notched apically [13]. This new species is also similar to *B. asiatica* (Brodsky, 1930) from Russia, Afghanistan, Pakistan, Sri Lanka, and India, but it can be separated from the latter by the light brown 2nd to 7th sterna with pale stripes laterally, and the round and blunt dorsal carina. In *B. asiatica*, the 2nd to 7th sterna are dark brown, and the dorsal carina has a very pointed and downcurved tip which is almost parallel to the plate sometimes [13].

#### 3.2.2. *Blepharicera* *dushanzica* sp. nov.

urn:lsid:zoobank.org:act:7FA1EAD7-A1C9-486C-90A3-89B2BD7A3308

**Type material. *Holotype*:** male (CAU), China: Xinjiang Autonomous Region, Dushanzi district, Duku Highway (44°5′36″ N 84°44′59″ E, 1397 m), 2017.VII.25, Jinlong Ren. ***Paratypes*:** 4 males & 4 females (QAU), same data as holotype.

**Diagnosis.** Compound eye with dorsal division 1/3 of ventral division in male. Mesonotum brownish black with a brownish yellow median stripe at posterior 1/2. Scutellum brown. Episternum brown; epimeron yellow. Rs slightly shorter than r-m. Gonostylus slightly swollen and notched apically. Dorsal paramere with tip bilobed; dorsal carina with tip very point, spike-like. Medial depression of 8th sternite M-shaped in female. Genital fork X-shaped in female.

**Description. Male.** Body length 4.00–5.50 mm; wing length 4.50–6.00 mm, width 1.50–2.00 mm.

**Figure 5:**
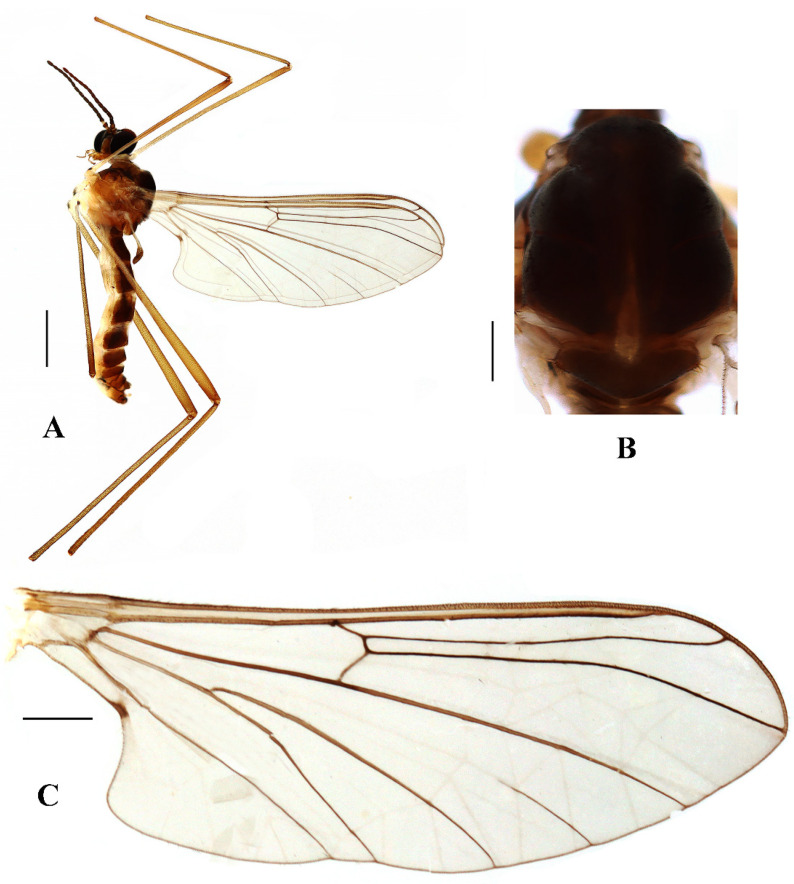
*Blepharicera dushanzica***sp. nov.** (male holotype). (**A**) habitus of male, lateral view (**B**) thorax, dorsal view (**C**) wing. Scale bars: 1.0 mm (**A**), 0.25 mm (**B**), 0.5 mm (**C**).

**Figure 6:**
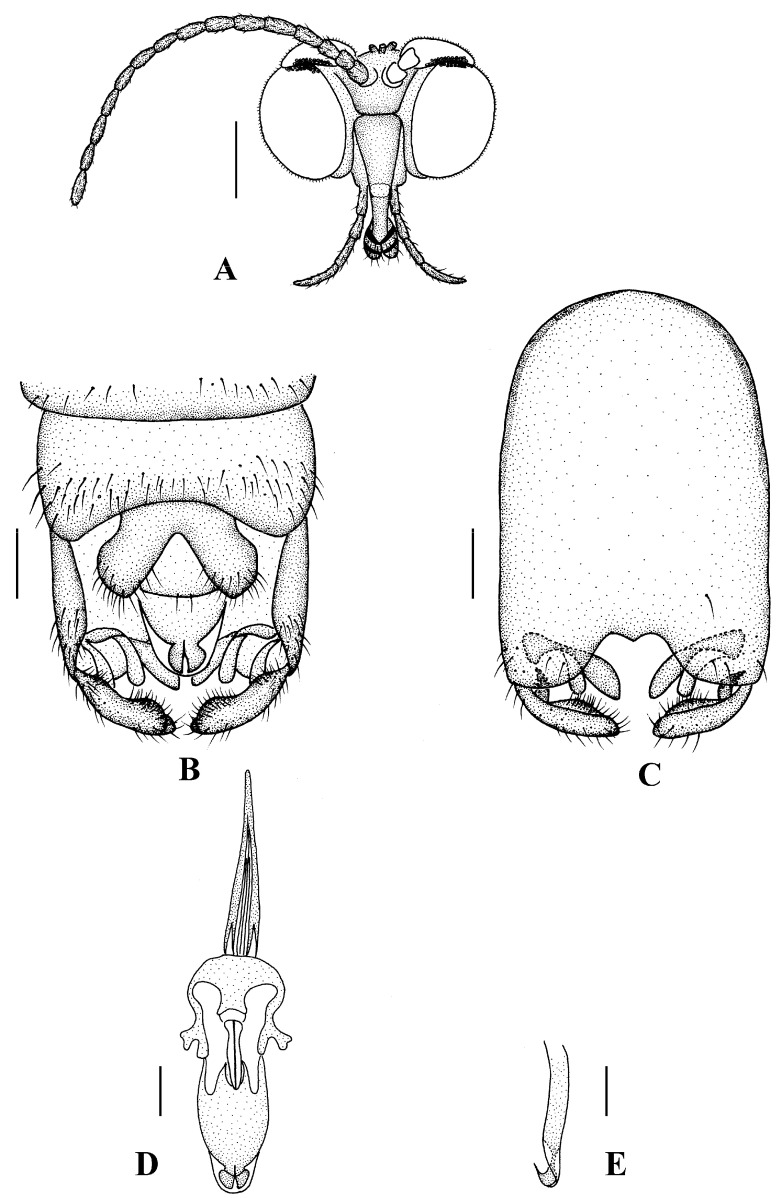
*Blepharicera dushanzica***sp. nov.** (male holotype). (**A**) male head, frontal view (**B**) male genitalia, dorsal view (**C**) male genitalia, ventral view (**D**) aedegal complex, dorsal view (**E**) tip of dorsal paramere, lateral view. Scale bars: 0.25 mm (**A**), 0.1 mm (**B**–**E**).

Head (Figure 5A and Figure 6A) uniformly brownish black with black hairs. Compound eyes dichoptic, interocular ridge absent; each compound eye divided, callis oculi absent; dorsal division contiguous with ventral division, 1/3 of ventral division; dorsal division with 18–20 rows of ommatidia, ommatidia red-orange, larger in diameter, with ommatrichia; ventral division black with ommatrichia. Ocelli brownish black. Scape and pedicel oval, brown with brownish black hairs; first flagellomere constricted at base, widening at apex, basal 1/2 pale, apical 1/2 brownish black, with brownish-black hairs; other flagellomeres cylindrical, dark brown with brownish black hairs; ultimate flagellomere 1.3 times length of penultimate flagellomere. Clypeus rectangular, dark brown, as long as wide; labrum brownish yellow; labellum brownish yellow with brownish black hairs; proboscis about 0.65 times the length of head width. Palpus with five segments, 1st segment almost invisible; 2nd to 4th segments cylindrical, yellow with brownish black hairs; 5th segment slender, brownish yellow with brownish black hairs; relative length of distal four segments as 1.1:1.0:1.1:1.8.

Thorax (Figure 5B). Pronotum and propleuron brown without hairs. Mesonotum brownish black with a brownish yellow median stripe at posterior 1/2; scutellum brown with numerous hairs grouped at the posterolateral corner; metanotum brown; episternum brown; epimeron yellow. The relative length of the femur, tibiae, and 1st to 5th tarsomeres in the fore leg as 15:10.5:7.8:3.6:2.2:1:1, in the mid-leg as 13.8:10.5:6.2:2.7:1.8:1:1, in the hind leg as 15.5:12.7:5:1.2:1:0.8:1. Fore coxa pale with basal 1/2 light brown, with light brown hairs; mid coxa with an appendage, pale with brownish black hairs; hind coxa pale with brownish black hairs; trochanters pale, anterior margin with black spot apically, with brownish-black hairs; femora pale basally and gradually darkened to brown apically, with brownish-black hairs; tibiae brown with brownish black hairs; tarsomeres brown yellow with brownish black hairs; claw brownish yellow. Tibial spurs 0–0–0. Wing (Figure 5C) slightly brown apically, sc light brown; veins brown. Sc rudimentary, not ending at the base of Rs; Rs slightly shorter than r-m; R_4_ wavy, the length from the end of R_1_ to end of R_4_ shorter than the length from the end of R_4_ to end of R_5_; r-m slightly curved apically, included angle between r-m and Rs less than 90 degrees; the length from the end of M_1_ to end of M_2_ as long as the length from the end of M_2_ to end of CuA_1_. The base of halter is pale, the apex of halter light brown with brownish black hairs.

Abdomen. First and 2nd terga brown, 3rd to 7th terga dark brown with basal 1/3 brown, 8th tergum dark brown; sterna pale; abdomen with brownish black hairs. Male genitalia (Figure 6B–E) brown with basal 2/3 hypandrium pale. Epandrium trapeziform, posterior margin concave, with several brown hairs. Cercus triangular, inner margin straight, with several brown hairs; anal cone straight. Gonostylus slightly swollen and notched apically, outer side with a wide triangular lobe folded ventrally, with hairs. Gonocoxal lobe bifurcated, outer gonocoxal lobe transparent, rod-shaped, slightly curved; inner gonocoxal lobe transparent, fusiform, nearly straight, broader than outer gonocoxal lobe. Hypandrium rectangular, twice as long as wide, slightly narrow basally, posterior margin concave, with several brown hairs. Aedeagus slender, curved backward; dorsal paramere with tip bilobed, posterior margin round; dorsal carina clearly visible, tip very point, spike-like.

**Female**. Body length 6.50–7.50 mm, wing length 7.00–8.00 mm, wing width 2.20–2.50 mm.

**Figure 7:**
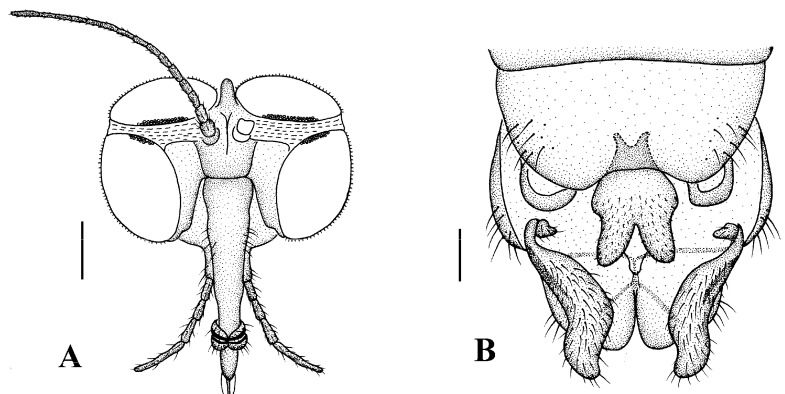
*Blepharicera dushanzica***sp. nov.** (female paratype). (**A**) female head, frontal view (**B**) female terminalia, ventral view. Scale bars: 0.25 mm (**A**), 0.10 mm (**B**).

Head (Figure 7A). Compound eyes subholoptic, interocular ridge present; each compound eye divided, callis oculi present; dorsal division separated from ventral division, as large as ventral division; dorsal division with about 20 rows of ommatidia, ommatidia red-orange, larger in diameter, with ommatrichia; ventral division black with ommatrichia. Scape and pedicel oval, light brown with brownish black hairs; first flagellomere constricted at base, widening at apex, basal 1/2 pale, apical 1/2 brownish black, with brownish-black hairs; other flagellomeres cylindrical, tapering apically, brownish black with brownish black hairs; ultimate flagellomere 1.72 times length of penultimate flagellomere. Labrum dark brown; labellum brownish yellow with brownish black hairs; mandible brownish yellow; proboscis about 0.81 times length of head width. Palpus with five segments, 1st segment almost invisible, yellow with brownish black hairs; 2nd to 4th segments cylindrical, yellow with brownish black hairs; 5th segment slender, cylindrical, yellow with brownish black hairs; relative length of distal four segments as 1.1:1.0:1.3:2.6. Tibial spurs 0–0–2. Terminalia (Figure 7B): 8th sternite approximately trapezoidal, bilobate posteriorly, medial depression M-shaped, with six hairs laterally; genital fork X-shaped; hypogynial plate broad basally, bilobate posteriorly, with round bulge laterally, each lobe round apically, intervalvular area V-shaped, with short hairs posteriorly; epiproct conical with two prominent hairs apically; spermathecae three in number.

**Distribution.** Currently known only from China (Xinjiang).

**Etymology.** The specific name refers to the type locality Dushanzi.

**Remarks.** This new species is very similar to *B. indica* from Afghanistan, Pakistan, Sri Lanka, and India but can be separated by the dorsal division being contiguous with ventral division and being about 1/3 of ventral division, the gonostylus being broad and distinctly notched apically, the dorsal paramere with tip bilobed, and the dorsal carina with tip very point, spike-like. In *B. indica*, the dorsal division is separated from ventral division by a distinct suture and is as large as ventral division, the gonostylus is distinctly slender and not distinctly notched apically, the tip of the dorsal paramere is not bilobed, and the dorsal carina is simple finger-like [13]. This new species is also similar to *B. asiatica* from Russia, Afghanistan, Pakistan, Sri Lanka, and India, but it can be separated from the latter by the brown scutellum, the pale sterna of the abdomen, and the dorsal paramere with tip bilobed. In *B. asiatica*, the scutellum is dark brown, the sterna of the abdomen are dark brown, and the dorsal paramere is not bilobed [13].

#### 3.2.3. *Blepharicera* *nigra* sp. nov.

urn:lsid:zoobank.org:act:D15D6F7C-8381-4EFF-93FE-B5F5F857F2DC

**Type material. *Holotype*:** male (CAU), China: Yunnan Province, Gejiu County, Lvshuihe Forest Park (505m), 2019.III.30, Liang Wang. ***Paratypes*:** 8 males & 10 females (QAU), same data as holotype; 5 males & 6 females (QAU), China: Yunnan Province, Gejiu County, Lvshuihe Forest Park (505m), 2019. III.30, Xin Li.

**Diagnosis.** Compound eye with dorsal division 1/20 of ventral division in male. Mesonotum brownish black. Scutellum brownish black. Mesopleuron mostly dark brown, except katepimeron light brown. Rs 1.2 times as long as r-m. Gonostylus slightly swollen and notched deeply at tip. Dorsal paramere with tip U-shaped; dorsal carina with tip round and blunt. Medial depression of 8th sternite U-shaped in female. Genital fork X-shaped in female.

**Description. Male.** Body length 4.25–4.50 mm; wing length 4.00–4.30 mm, width 1.50–1.60 mm.

**Figure 8:**
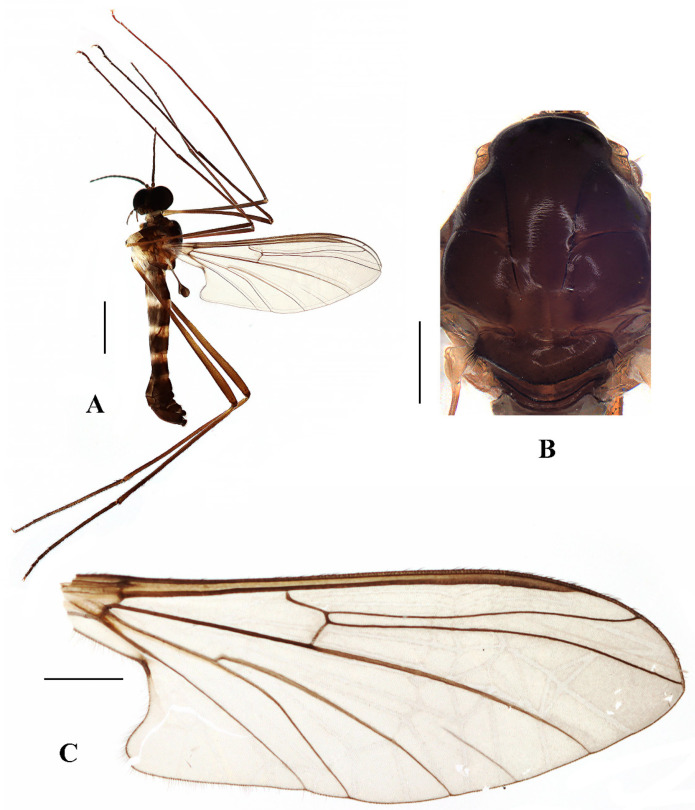
*Blepharicera nigra***sp. nov.** (male holotype). (**A**) habitus of male, lateral view (**B**) thorax, dorsal view (**C**) wing. Scale bars: 1.0 mm (**A**), 0.25 mm (**B**), 0.5 mm (**C**).

**Figure 9:**
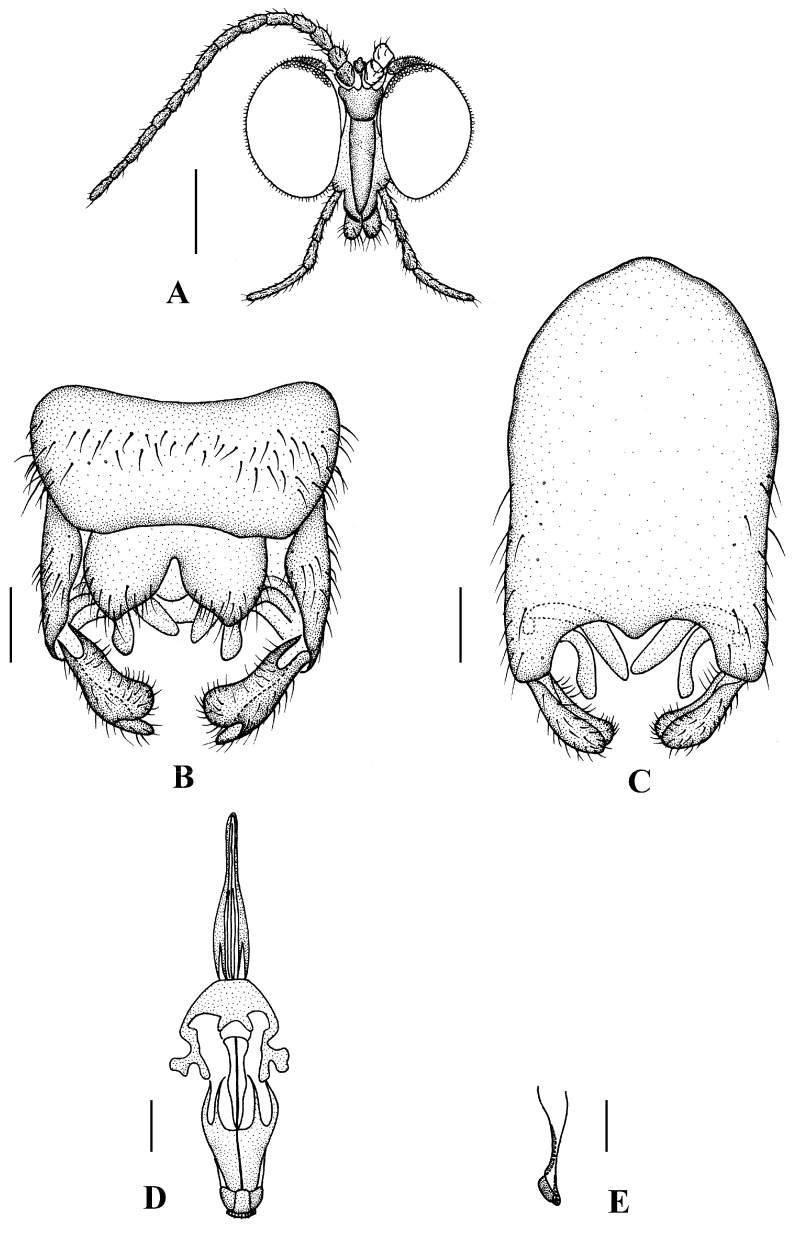
*Blepharicera nigra***sp. nov.** (male holotype). (**A**) male head, frontal view (**B**) male genitalia, dorsal view (**C**) male genitalia, ventral view (**D**) aedegal complex, dorsal view (**E**) tip of dorsal paramere, lateral view. Scale bars: 0.25 mm (**A**), 0.1 mm (**B**–**E**).

Head (Figure 8A and Figure 9A) uniformly black with black hairs. Compound eyes dichoptic, interocular ridge absent; each compound eye divided, callis oculi absent; dorsal division contiguous with ventral division, 1/20 of ventral division; dorsal division with 6–7 rows of ommatidia, ommatidia brownish yellow, larger in diameter, with ommatrichia; ventral division black with ommatrichia. Ocelli brownish yellow. Scape and pedicel oval, black with black hairs; first flagellomere constricted at base, widening at apex, basal 1/2 brownish yellow, apical 1/2 brownish black, with black hairs; other flagellomeres cylindrical, brownish black with brownish black hairs; ultimate flagellomere 1.5 times length of penultimate flagellomere. Clypeus nearly rectangular, brownish black, twice as long as wide; labrum brownish yellow; labellum brownish yellow with brownish black hairs; proboscis about 0.52 times the length of head width. Palpus with five segments, 1st segment almost invisible; 2nd and 3rd segments cylindrical, brownish black with black hairs; 4th segment cylindrical, slightly swollen apically, brownish black with black hairs; 5th segment slender, brownish yellow with brownish black hairs; relative length of distal four segments as 1.0: 0.8: 1.0: 2.3.

Thorax (Figure 8B). Pronotum and propleuron brownish black without hairs. Mesonotum brownish black; scutellum brownish black with numerous hairs grouped at the posterolateral corner; metanotum brownish black; mesopleuron mostly brownish black, except katepimeron light brown. The relative length of the femur, tibiae, and 1st to 5th tarsomeres in the fore leg as 11:9.2:6.7:2.9:2:0.8:1, in mid leg as 12.5:9.5:6.5:2.3:2:0.8:1, in hind leg as 16.7:14.6:6.1:2:1.4:0.9:1. Fore coxa dark brown with brownish black hairs; mid coxa with an appendage, pale with brownish black hairs; hind coxa pale with brownish black hairs; trochanters pale, anterior margin with black spot apically, with brownish-black hairs; fore and mid femora brownish yellow basally and gradually darkened apically, with brownish-black hairs; hind femur yellowish brown basally, the middle area gradually darkened to dark brown and gradually lightened to brown apically, with brownish-black hairs; fore and mid tibiae dark brown with brownish black hairs; hind tibia brown with brownish black hairs; tarsomeres dark brown with brownish black hairs, hind tarsomere with a black long hair basally; claw brown. Tibial spurs 0–0–0. Wing (Figure 8C) slightly brown apically, sc brown; veins brown. Sc rudimentary, not ending at the base of Rs; Rs slightly curved basally, 1.2 times as long as r-m; R_4_ wavy, the length from the end of R_1_ to end of R_4_ shorter than the length from the end of R_4_ to end of R_5_; r-m straight, included angle between r-m and Rs less than 90 degrees; the length from the end of M_1_ to end of M_2_ as long as the length from the end of M_2_ to end of CuA_1_. The base of halter is pale, the apex of halter brownish black, with brownish-black hairs.

Abdomen. First tergum brownish black with middle area pale, 2nd tergum brownish black, 3rd to 5th terga brownish black with basal 1/3 pale, 6th to 8th terga brownish black; 1st to 5th sterna brownish black with basal 1/3 pale, 6th and 7th terga brownish black; abdomen with brownish black hairs. Male genitalia (Figure 9B–E) brownish black. Epandrium trapeziform, posterior margin concave, with several brownish-black hairs. Cercus triangular, inner margin bulge, with several brownish-black hairs; anal cone round. Gonostylus slightly swollen and notched deeply at the tip, outer side with a wide triangular lobe folded ventrally, with hairs. Gonocoxal lobe bifurcated, outer gonocoxal lobe transparent, rod-shaped and curved, round apically; inner gonocoxal lobe transparent, rod-shaped, slightly curved, point apically. Hypandrium rectangular, twice as long as wide, slightly narrow basally, posterior margin concave, with several brownish-black hairs. Aedeagus slender, curved backward; dorsal paramere with tip U-shaped, posterior margin round; dorsal carina clearly visible, tip round and blunt.

**Female**. Body length 4.50–6.00 mm, wing length 5.00–7.50 mm, wing width 1.80–2.20 mm.

**Figure 10:**
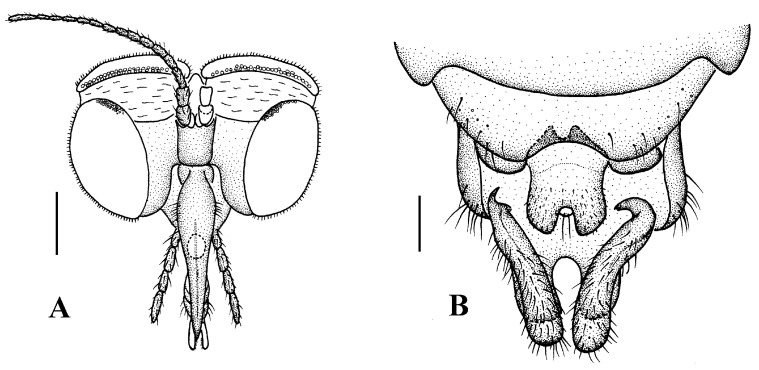
*Blepharicera nigra***sp. nov.** (female paratype). (**A**) female head, frontal view (**B**) female terminalia, ventral view. Scale bars: 0.25 mm (**A**), 0.10 mm (**B**).

Head (Figure 10A). Compound eyes subholoptic, interocular ridge present; each compound eye divided, callis oculi present; dorsal division separated from ventral division, as large as ventral division; dorsal division with about 14 rows of ommatidia, ommatidia red-orange, larger in diameter, with ommatrichia; ventral division black with ommatrichia. Scape oval, black with black hairs; pedicel cylindrical, black with black hairs; first flagellomere cylindrical, constricted at base, brownish black with basal 1/5 brown, with black hairs; other flagellomeres cylindrical, tapering apically, brownish black with black hairs; ultimate flagellomere twice length of penultimate flagellomere. Labrum brownish black; labellum pale with brownish black hairs; mandible present, brownish yellow; proboscis about 0.73 times length of head width. Palpus with five segments, 1st segment almost invisible, brown with brownish black hairs; 2nd to 5th segments cylindrical, brownish black with brownish black hairs; relative length of distal four segments as 1.0:1.2:1.2:1.6. Tibial spurs 0–0–2. Terminalia (Figure 10B): 8th sternite approximately trapezoidal, bilobate posteriorly, medial depression U-shaped, with several hairs laterally; genital fork X-shaped; hypogynial plate broad basally, bilobate posteriorly, each lobe round apically, intervalvular area U-shaped, with short hairs posteriorly; epiproct round with two prominent hairs apically; spermathecae three in number.

**Distribution.** Currently known only from China (Yunnan).

**Etymology.** The specific name is from Latin nigia (adjective, feminine, meaning black), referring to the body color mostly being brownish black to black.

**Remarks.** This new species is very similar to *B. japonica* (Kitakami, 1931) from Japan but can be separated by the mandible being present in females, the mid coxa with an appendage, the mid trochanter is not modified, and the dorsal paramere with a tip U-shaped medially. In *B. japonica*, the mandible is absent in females, the mid coxa has no appendage, the mid trochanter is modified, and the tip of the dorsal paramere is vestigial arrow-head-pattern [13,36]. This new species is also similar to *B. parva* Zwick *et* Arefina, 2005 from the Russian Far East but can be separated by the cercus being tapered posteriorly, the gonostylus being notched deeply at the tip but not bifurcated, and the rod-shaped inner gonocoxal lobe. In *B. parva*, the cercus is round, the gonostylus is deeply bifurcated, and the inner gonocoxal lobe is blade-like [37].

#### 3.2.4. *Blepharicera* *xinjiangica* sp. nov.

urn:lsid:zoobank.org:act:218C6007-4FAC-4190-AE45-BBF36A78EB00

**Type material. *Holotype*:** male (CAU), China: Xinjiang Autonomous Region, Dushanzi district, Duku Highway (44°5’36’’ N 84°44’59’’ E, 1397 m), 2017.VII.25, Jinlong Ren. ***Paratypes*:** 5 males (QAU), same data as holotype; 1 male & 2 females (QAU), China: Xinjiang Autonomous Region, Zhaosu County, Qiongbola Forest Park (43°26’3’’ N 81°1’13’’ E, 1976 m), 2017.VII.30, Bing Zhang; 2 females (QAU), China: Xinjiang Autonomous Region, Emin County, Hudie Valley (46°56’38’’ N 84°40’38’’ E, 1457 m), 2017.VIII.2, Bing Zhang.

**Diagnosis.** Compound eye with dorsal division 1/3 of ventral division in male. Mesonotum dark brown with posterior margin yellow, posterior 1/2 with the extensive light brown middle area, and a brownish yellow narrow median stripe. Scutellum light brown with anterior 1/3 yellow. Episternum light brown; epimeron yellow. Rs longer than r-m. Gonostylus slightly swollen and notched apically. Dorsal paramere with tip arrowhead-shaped; dorsal carina clearly visible, tip point. Medial depression of 8th sternite M-shaped in female. Genital fork X-shaped in female.

**Description. Male.** Body length 4.50–6.00 mm; wing length 5.00–6.50 mm, width 2.00–2.20 mm.

**Figure 11:**
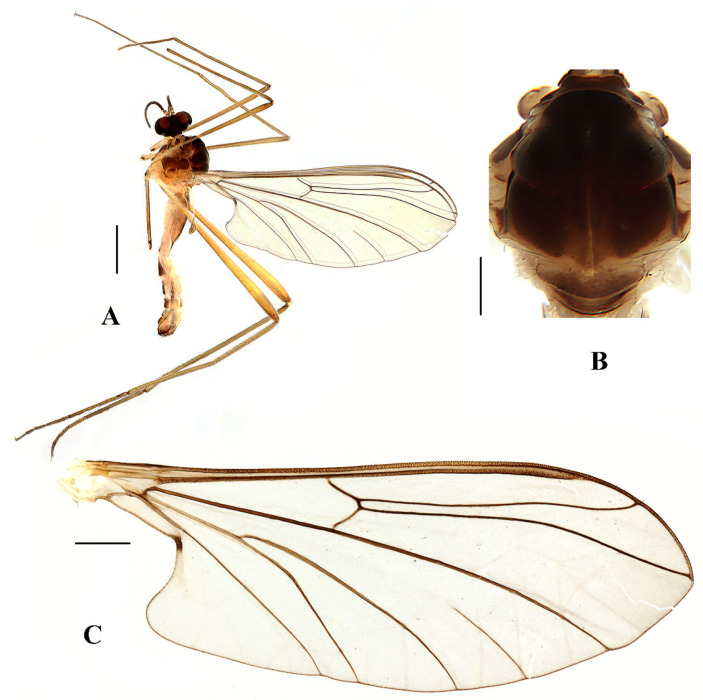
*Blepharicera xinjiangica***sp. nov.** (male holotype). (**A**) habitus of male, lateral view (**B**) thorax, dorsal view (**C**) wing. Scale bars: 1.0 mm (**A**), 0.25 mm (**B**), 0.5 mm (**C**).

**Figure 12:**
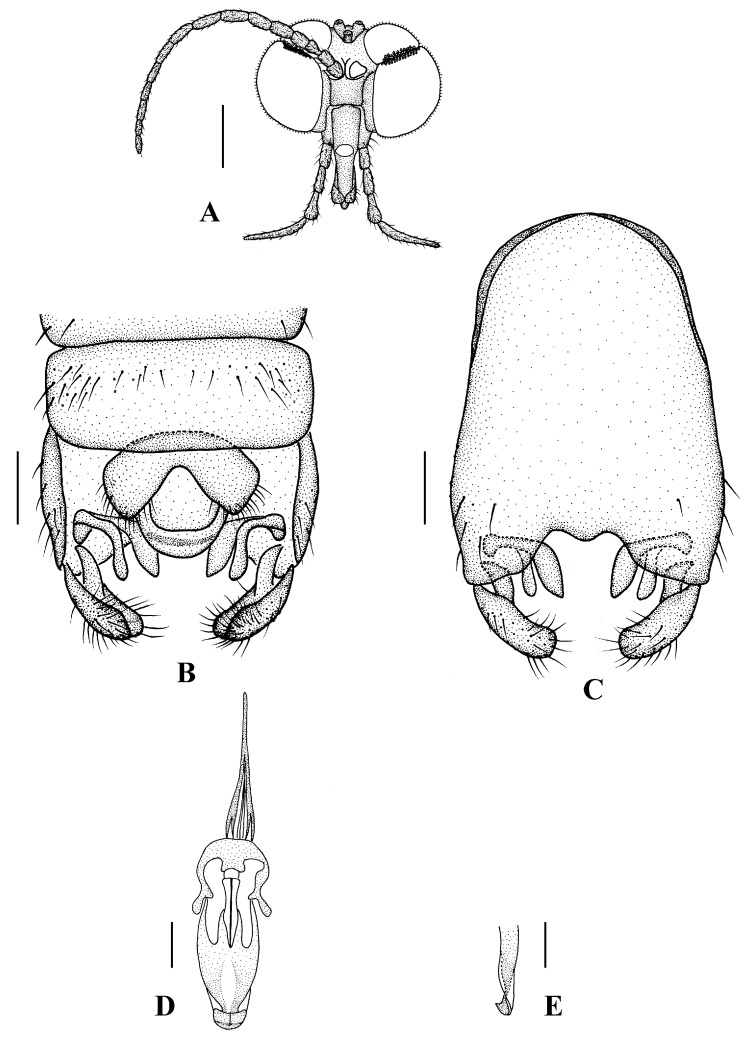
*Blepharicera xinjiangica***sp. nov.** (male holotype). (**A**) male head, frontal view (**B**) male genitalia, dorsal view (**C**) male genitalia, ventral view (**D**) aedegal complex, dorsal view (**E**) tip of dorsal paramere, lateral view. Scale bars: 0.25 mm (**A**), 0.1 mm (**B**–**E**).

Head (Figure 11A and Figure 12A) uniformly brownish black with black hairs. Compound eyes dichoptic, interocular ridge absent; each compound eye divided, callis oculi absent; dorsal division contiguous with ventral division, 1/3 of ventral division; dorsal division with 18–20 rows of ommatidia, ommatidia red-orange, larger in diameter, with ommatrichia; ventral division black with ommatrichia. Ocelli brownish black. Scape and pedicel oval, light brown with brownish black hairs; first flagellomere constricted at base, widening at apex, basal 1/2 pale, apical 1/2 dark brown, with brownish-black hairs; other flagellomeres cylindrical, dark brown with brownish black hairs; ultimate flagellomere 1.3 times length of penultimate flagellomere. Clypeus rectangular, dark brown, as long as wide; labrum brownish yellow; labellum brownish yellow with brown hairs; proboscis about 0.57 times the length of head width. Palpus with five segments, 1st segment almost invisible; 2nd and 3rd segments cylindrical, yellow with brownish black hairs; 4th segment cylindrical, slightly swollen apically, brownish yellow with brownish black hairs; 5th segment slender, brownish yellow with brownish black hairs; relative length of distal four segments as 1.0:1.0:1.1:2.6.

Thorax (Figure 11B). Pronotum and propleuron brown without hairs. Mesonotum dark brown with posterior margin yellow, posterior 1/2 with the extensive light brown middle area and a brownish yellow narrow median stripe; scutellum light brown with anterior 1/3 yellow, numerous hairs grouped at posterolateral corner of scutellum; metanotum light brown; episternum light brown; epimeron yellow. The relative length of the femur, tibiae, and 1st to 5th tarsomeres in fore leg as 16:11:8:4:2.5:1:1, in mid leg as 14:10.8:6.5:3:1.8:1:1, in hind leg as 15.7:12.7:5:1.3:1:0.7:1. Fore coxa pale with basal 1/2 light brown, with light brown hairs; mid coxa with an appendage, pale with brownish black hairs; hind coxa pale with brownish black hairs; trochanters pale, anterior margin with black spot apically, with brownish-black hairs; femora brownish yellow with light brown ring apically, with brownish-black hairs; tibiae brownish yellow with brownish black hairs; tarsomeres brownish yellow with brownish black hairs; claw brownish yellow. Tibial spurs 0–0–0. Wing (Figure 11C) slightly brown apically, sc light brown; veins brown. Sc rudimentary, not ending at the base of Rs; Rs slightly curved basally, longer than r-m; R_4_ wavy, the length from the end of R_1_ to end of R_4_ shorter than the length from the end of R_4_ to end of R_5_; r-m straight, included angle between r-m and Rs less than 90 degrees; the length from the end of M_1_ to end of M_2_ longer than the length from the end of M_2_ to end of CuA_1_. The base of halter is pale, the apex of halter light brown with brownish black hairs.

Abdomen. First tergum brown with middle area pale, 2nd to 6th terga brown with basal 1/3 light brown, 7th and 8th terga brown; sterna pale; abdomen with brownish black hairs. Male genitalia (Figure 12B–E) brown with basal 1/3 hypandrium pale. Epandrium rectangular, with several brown hairs. Cercus triangular, inner margin slightly bulge, with several brown hairs; anal cone straight. Gonostylus slightly swollen and notched apically, outer side with a wide triangular lobe folded ventrally, with hairs. Gonocoxal lobe bifurcated, outer gonocoxal lobe transparent, rod-shaped, slightly curved; inner gonocoxal lobe transparent, fusiform, slightly curved, broader than outer gonocoxal lobe. Hypandrium rectangular, twice as long as wide, slightly narrow basally, posterior margin concave, with several brown hairs. Aedeagus slender, curved backward; dorsal paramere with tip arrowhead-shaped and posterior margin round; dorsal carina clearly visible, tip point.

**Female**. Body length 6.00–7.00 mm, wing length 7.00–7.50 mm, wing width 2.25–2.50 mm.

**Figure 13:**
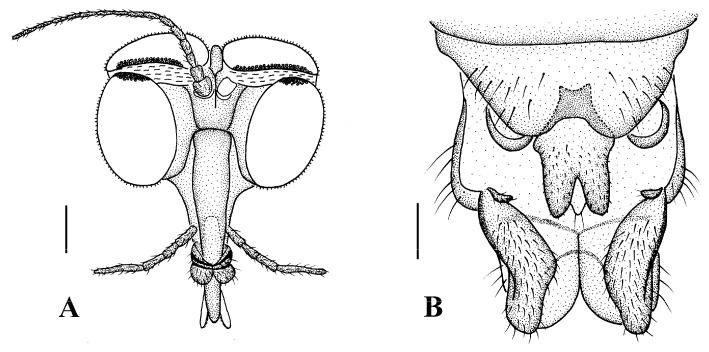
*Blepharicera xinjiangica***sp. nov.** (female paratype). (**A**) female head, frontal view (**B**) female terminalia, ventral view. Scale bars: 0.25 mm (**A**), 0.10 mm (**B**).

Head (Figure 13A). Compound eyes subholoptic, interocular ridge present; each compound eye divided, callis oculi present; dorsal division separated from ventral division, as large as ventral division; dorsal division with about 20 rows of ommatidia, ommatidia red-orange, larger in diameter, with ommatrichia; ventral division black with ommatrichia. Scape and pedicel oval, light brown with brownish black hairs; first flagellomere constricted at base, widening at apex, basal 1/2 pale, apical 1/2 brownish black, with brownish-black hairs; other flagellomeres cylindrical, tapering apically, brownish black with brownish black hairs; ultimate flagellomere 1.54 times length of penultimate flagellomere. Labrum brown; labellum brownish yellow with brownish black hairs; mandible brownish yellow; proboscis about 0.86 times length of head width. Palpus with five segments, 1st segment almost invisible, yellow with brownish black hairs; 2nd to 4th segments cylindrical, yellow with brownish black hairs; 5th segment slender, cylindrical, yellow with brownish black hairs; relative length of distal four segments as 1.0:0.8:1.0:2.2. Tibial spurs 0–0–2. Terminalia (Figure 13B): 8th sternite approximately trapezoidal, bilobate posteriorly, medial depression M-shaped, with six hairs laterally; genital fork X-shaped; hypogynial plate broad basally, bilobate posteriorly, with triangular bulge laterally, each lobe round apically, intervalvular area V-shaped, with short hairs posteriorly; epiproct conical with two prominent hairs apically; spermathecae three in number.

**Distribution.** Currently known only from China (Xinjiang).

**Etymology.** The specific name refers to the type locality Xinjiang.

**Remarks.** This new species is very similar to *B. asiatica* from Russia, Afghanistan, Pakistan, Sri Lanka, and India, but it can be separated from the latter by the mesonotum being dark brown with a yellow posterior margin, extensive light brown middle area at the posterior 1/2 and a brownish yellow narrow median stripe, the scutellum being light brown with yellow anterior 1/3, the pale sterna of the abdomen, the dorsal paramere with tip arrowhead-shaped, and the dorsal carina being not very pointed and not downcurved. In *B. asiatica*, the mesonotum and scutellum are dark brown, the sterna of the abdomen are dark brown, the tip of the dorsal paramere is conical, and the dorsal carina has a very pointed and downcurved tip that is almost parallel to plate sometimes [13]. This new species is also very similar to *B. balangshana* Zhang *et* Kang, 2022 from China but can be separated by the dorsal division being 1/3 of ventral division, the inner gonocoxal lobe being fusiform and the dorsal carina being clearly visible with point tip. In *B. balangshana*, the dorsal division is as large as the ventral division, the inner gonocoxal lobe is rod-shaped, and the dorsal carina has a perpendicular tip [13].

#### 3.2.5. Keys to Chinese *Blepharicera* Species

**Key for adult males**

1. Dorsal division of compound eye large, at least 1/3 of ventral division (Figure 6A and Figure 12A) ... 2

-Dorsal division of compound eye small, at most 1/10 of ventral division (Figure 3A and Figure 9A) ... 7

2. Gonostylus bifurcated … 3

-Gonostylus notched apically but not bifurcated (Figure 3B, Figure 6B, Figure 9B, and Figure 12B) … 4

3. Ultimate flagellomere shorter than penultimate flagellomere; Rs 1.5 times as long as r-m; ventral branch of gonostylus glabrous [15] ... *B. taiwanica* (Kitakami, 1937)

-Ultimate flagellomere longer than penultimate flagellomere; Rs as long as or slightly longer than r-m; ventral branch of gonostylus with two tufts of short dense setae [18] ... *B. macropyga* Zwick, 1990

4. Epandrium semicircular; cercus semi-elliptical; gonostylus with a semicircular inside lobe near base [18] ... *B. hainana* Kang *et* Yang, 2014

-Epandrium trapeziform or rectangular; cercus triangular; gonostylus without a semicircular inside lobe near base … 5

5. Dorsal division of compound eye as large as ventral division; inner gonocoxal lobe rod-shaped ... *B. balangshana* Zhang *et* Kang, 2022

-Dorsal division of compound eye 1/3 of ventral division; inner gonocoxal lobe fusiform … 6

6. Wing with Rs slightly shorter than r-m (Figure 5C); mesonotum brownish black with a brownish yellow median stripe at posterior 1/2; scutellum uniformly brown; epandrium trapeziform (Figure 5B); dorsal paramere with tip bilobed (Figure 6D); dorsal carina with tip very point, spike-like (Figure 6E) … *B. dushanzica* sp. nov.

-Wing with Rs longer than r-m (Figure 11C); mesonotum dark brown with posterior margin yellow, posterior 1/2 with the extensive light brown middle area and a brownish yellow narrow median stripe; scutellum light brown with anterior 1/3 yellow (Figure 11B); epandrium rectangular; dorsal paramere with tip arrowhead-shaped (Figure 12D); dorsal carina with the tip not very pointed (Figure 12E) … *B. xinjiangica* **sp. nov.**

7. Cercus triangular (Figure 3B, Figure 6B, Figure 9B, and Figure 12B) ... 8

-Cercus semicircular or semi-elliptical ... 13

8. Outer gonocoxal lobe straight ... 9

-Outer gonocoxal lobe S-shaped ... 12

9. Abdomen with second to 7th sterna uniformly dark brown; dorsal carina pointed and downcurved [13] … *B*. *asiatica* (Brodsky, 1930)

-Abdomen with second to 7th sterna with the basal or middle pale area; dorsal carina flat or round … 10

10. Body length 3.00–4.00 mm; wing with Rs as long as r-m [20] … *B. xizangica* Kang, Zhang *et* Yang, 2022

-Body length 4.00–5.00 mm; wing with Rs longer than r-m … 11

11. Wing with Rs 1.5 times as long as r-m (Figure 2C); dorsal paramere with tip arrowhead-shaped (Figure 3D) … *B. beishanica* sp. nov.

-Wing with Rs 1.2 times as long as r-m (Figure 8C); dorsal paramere with tip U-shaped (Figure 9D) … *B. nigra* **sp. nov.**

12. Ultimate flagellomere shorter than penultimate flagellomere; dorsal branch of gonostylus broader than ventral branch; inner gonocoxal lobe fusiform; dorsal carina subtle ... *B. kongsica* Zhang *et* Kang, 2022

-Ultimate flagellomere longer than penultimate flagellomere; dorsal branch of gonostylus as broad as ventral branch; inner gonocoxal lobe digitiform; dorsal carina clearly visible ... *B. gengdica* Zhang *et* Kang, 2022

13. Mid coxa with a conical projection, conical projection about half as long as trochanter and densely with stiff black bristles towards tip [16] ... *B. yamasakii* (Kitakami, 1950)

-Mid coxa without projection like above ... 14

14. Posterior margin of epandrium not distinctly concaved medially; cercus semicircilar; gonostylus bifurcated and strongly notched apically [18] ... *B. dimorphops* Alexander, 1953

-Posterior margin of epandrium concave medially, V-shaped; cercus semi-elliptical; gonostylus not bifurcated and slightly notched apically [18] ... *B. hebeiensis* Kang *et* Yang, 2014

**Key for fourth instar larvae**

1. Feeler entirely absent; 7th segment without appendage; caudal appendage absent; suckers rather small (Kitakami, 1950: plate V, figure 55) [16] … *B. yamasakii* (Kitakami, 1950)

-A rudimentary posterior feeler present on 4th to 6th abdominal segments; 7th segment with appendage; caudal appendage present; suckers rather large … 2

2. Dorsal integument with densely long and curved whitish hairs (Kitakami, 1937: plate II, figure 18); ventral integument with short setae sparse (Kitakami, 1937: plate II, figure 19); a thoracic spot not fully developed; 1st to 5th abdominal segments with single wart; 7th abdominal segment with a pair of short distally chitinized appendages; caudal appendage short (Kitakami, 1937: plate II, figure 18; Kitakami, 1941: plate I, figure 13) [15,38] … *B. taiwanica* (Kitakami, 1937)

-Dorsal integument with sparsely setaceous (Kitakami, 1937: plate II, figure 20); ventral integument glabrous (Kitakami, 1937: plate II, figure 21); thoracic spot V-shaped; claws large and elongated; 2nd to 4th abdominal segments with single sharp dorsal thorn; 7th abdominal segment with a pair of elongated strongly chitinized appendages; caudal appendage elongated (Kitakami, 1937: plate II, figure 20) [15,38] … *B. uenoi* (Kitakami, 1937)

### 3.3. Distribution of Chinese Blepharicera

**Figure 14:**
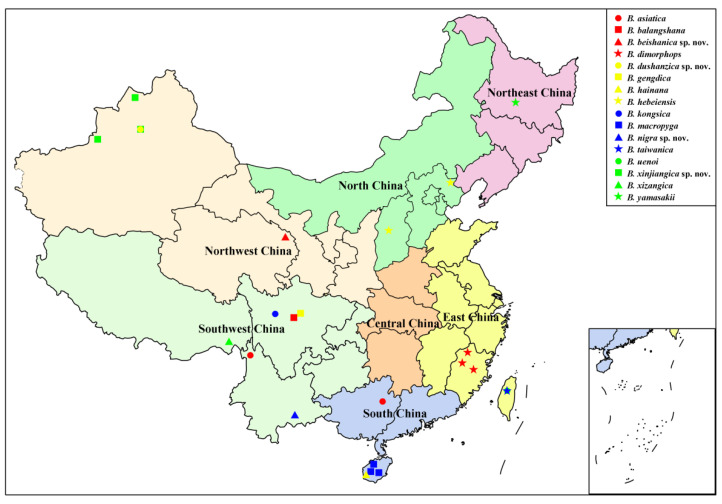
Distribution map of *Blepharicera* in China.

Based on a review of literature [13,15,16,17,18,19,20] and data in this study, a total of 16 *Blepharicera* species have been known to be distributed in China. Species records with detailed distribution data, as shown in Table 3, indicate that the genus *Blepharicera* is widely distributed in China, with distribution in six of the seven Chinese regions. Southwest China is the species richest region with six species, followed by East China, Northwest China, and South China with three species respectively. North China and Northeast China have the least number of species with only one species respectively, while no *Blepharicera* species have been found in Central China (Figure 14). The Chinese *Blepharicera* fauna is composed of exclusively Palaearctic or Oriental taxa with the exception of *B. asiatica*, which is widely distributed in the Palaearctic and Oriental regions. Current distribution data show that 15 species are restricted to China and can be considered Chinese endemics, and some of them have a small area of distribution, occurring in just one or a few sites.

## 4. Discussion

In molecular analysis, the smallest interspecific genetic distance is recorded between *B. dushanzica* **sp. nov.** and *B. xinjiangica* **sp. nov.**, ranging from 5.4% to 6.2%. However, the two species are represented by well-separated clades with robust support in each tree: the sequences of four *B. dushanzica* specimens from the same location (Duku Highway, Dushanzi County, Xinjiang) gather into one clade, while the sequences of three *B. xinjiangica* specimens from the same location with *B. dushanzica* and two *B. xinjiangica* specimens from other location (Qiongbola Forest Park, Zhaosu County, Xinjiang) gather into another clade. In addition, the taxa proposed are further supported by distinct morphological characters. The species *B. dushanzica* can be recognized by the mesonotum being brownish black with a brownish yellow median stripe at posterior 1/2, the brown scutellum, and the wing with Rs being slightly shorter than r-m. In *B. xinjiangica*, the mesonotum is dark brown with a yellow posterior margin, extensive light brown middle area at posterior 1/2 and a brownish yellow narrow median stripe, the scutellum is light brown with yellow anterior 1/3, and the vein Rs is longer than r-m. The two species can also be easily separated by the details of the male genitalia. Therefore, the small genetic distance could be attributed to recent speciation.

With our efforts, the number of *Blepharicera* species in China has increased from five to 16 in the past decade [18,19,20], including four new species in this study. However, larvae of only three species are known, one of which was described only based on larvae. Breeding net-winged midges is difficult, and if the larvae cannot be successfully raised to adults, it is almost impossible to match the larvae and adults based on morphology. In addition, *Blepharicera* species are usually sexually dimorphic, which also brings trouble to taxonomic research. However, DNA barcoding will be a powerful tool to enhance the larva-adult and female-male associations of the same species and fundamentally complement traditional taxonomy. Therefore, a comprehensive DNA barcode reference library for *Blepharicera* or even Blephariceridae is crucial for a more comprehensive understanding of their diversity, biology, distribution, monitoring, and so on in the future.

Geographical analysis shows that Southwest China is the species’ richest region, which may be related to the topography of the region. Southwest China has a wide area and complex terrain, including the Qinghai Tibet Plateau, the Hengduan Mountains, and the Sichuan Basin, and there are many rivers and lakes in the region, which is more conducive to the speciation of net-winged midges. In addition, this region is also considered a biodiversity hotspot [39]. It is worth mentioning that the genus *Blepharicera* is recorded from Northwest China for the first time in this study, with the species *B. beishanica* **sp. nov.**, *B. dushanzica* **sp. nov.** and *B. xinjiangica* **sp. nov.**, while three other Chinese regions, North China, Northeast China, and Central China, have very few *Blepharicera* species, with one species each or none at all. Although these results will be updated with the discovery of more new species and new distribution data in the future, the current results can still reveal the distribution of Chinese *Blepharicera* to a certain degree and provide direction for subsequent related research. This study may serve as a baseline for planning future work in China, and also in surrounding countries for which knowledge of the *Blepharicera* fauna is poor.

## 5. Conclusions

The molecular data (mt COI barcodes) used in this study has shown congruence with the morphological identification and greatly facilitated the positive male and female associations of the four new species. This initial attempt indicates the validity of COI barcodes for *Blepharicera* in delimiting species and contributes to the growing library of DNA barcodes of net-winged midges of the world. Molecular and morphological data show that the four populations examined represent members of the genus *Blepharicera* and constitute four new, distinct species. Our results will be useful in tackling taxonomic problems, understanding species distribution, and resolving nomenclature conflicts associated with *Blepharicera* species.

## Figures and Tables

**Table 1:** Information of *Blepharicera* specimens sequenced in this study with GenBank accession numbers of mt COI sequences.

Code	Species	Sex	Locality	Accession Number
BBlba_1	*B. balangshana* Zhang *et* Kang, 2022	male	CHINA: Sichuan, Xiaojin, Mount Balang (3281 m)	OP013293
BBlba_2	*B. balangshana* Zhang *et* Kang, 2022	male	CHINA: Sichuan, Xiaojin, Mount Balang (3281 m)	OP013294
BBlba_3	*B. balangshana* Zhang *et* Kang, 2022	male	CHINA: Sichuan, Xiaojin, Mount Balang (3281 m)	OP013295
BBlbe_1	*B. beishanica* **sp. nov.**	male	CHINA: Qinghai, Huzhu, Beishan Forest Farm (36°50′56″ N 101°56′47″ E, 2100 m)	OP013296
BBlbe_2	*B. beishanica* **sp. nov.**	female	CHINA: Qinghai, Huzhu, Beishan Forest Farm (36°50′56″ N 101°56′47″ E, 2100 m)	OP013297
BBlbe_3	*B. beishanica* **sp. nov.**	male	CHINA: Qinghai, Huzhu, Beishan Forest Farm (36°50′56″ N 101°56′47″ E, 2100 m)	OP013298
BBlbe_4	*B. beishanica* **sp. nov.**	male	CHINA: Qinghai, Huzhu, Beishan Forest Farm (36°50′56″ N 101°56′47″ E, 2100 m)	OP013299
BBlbe_5	*B. beishanica* **sp. nov.**	male	CHINA: Qinghai, Huzhu, Beishan Forest Farm (36°50′56″ N 101°56′47″ E, 2100 m)	OP013300
BBlbe_6	*B. beishanica* **sp. nov.**	male	CHINA: Qinghai, Huzhu, Beishan Forest Farm (36°50′56″ N 101°56′47″ E, 2100 m)	OP013301
BBldu_2	*B. dushanzica* **sp. nov.**	female	CHINA: Xinjiang, Dushanzi, Duku Highway (44°5′36″ N 84°44′59″ E, 1397 m)	OP013302
BBldu_3	*B. dushanzica* **sp. nov.**	male	CHINA: Xinjiang, Dushanzi, Duku Highway (44°5′36″ N 84°44′59″ E, 1397 m)	OP013303
BBldu_5	*B. dushanzica* **sp. nov.**	male	CHINA: Xinjiang, Dushanzi, Duku Highway (44°5′36″ N 84°44′59″ E, 1397 m)	OP013304
BBldu_7	*B. dushanzica* **sp. nov.**	female	CHINA: Xinjiang, Dushanzi, Duku Highway (44°5′36″ N 84°44′59″ E, 1397 m)	OP013305
BBlge_1	*B. gengdica* Zhang *et* Kang, 2022	male	CHINA: Sichuan, Wenchuan, Gengda, Fuyuan inn	OP013306
BBlko_1	*B. kongsica* Zhang *et* Kang, 2022	male	CHINA: Sichuan, Daofu, Kongse (2976 m)	OP013307
BBlko_2	*B. kongsica* Zhang *et* Kang, 2022	female	CHINA: Sichuan, Daofu, Kongse (2976 m)	OP013308
BBlko_3	*B. kongsica* Zhang *et* Kang, 2022	female	CHINA: Sichuan, Daofu, Kongse (2976 m)	OP013309
BBlko_4	*B. kongsica* Zhang *et* Kang, 2022	female	CHINA: Sichuan, Daofu, Kongse (2976 m)	OP013310
BBlko_5	*B. kongsica* Zhang *et* Kang, 2022	female	CHINA: Sichuan, Daofu, Kongse (2976 m)	OP013311
BBlni_1	*B. nigra* **sp. nov.**	male	CHINA: Yunnan, Gejiu, Lvshuihe Forest Park (505 m)	OP013312
BBlni_2	*B. nigra* **sp. nov.**	female	CHINA: Yunnan, Gejiu, Lvshuihe Forest Park (505 m)	OP013313
BBlni_3	*B. nigra* **sp. nov.**	male	CHINA: Yunnan, Gejiu, Lvshuihe Forest Park (505 m)	OP013314
BBlni_4	*B. nigra* **sp. nov.**	female	CHINA: Yunnan, Gejiu, Lvshuihe Forest Park (505 m)	OP013315
BBlni_5	*B. nigra* **sp. nov.**	male	CHINA: Yunnan, Gejiu, Lvshuihe Forest Park (505 m)	OP013316
BBlni_6	*B. nigra* **sp. nov.**	male	CHINA: Yunnan, Gejiu, Lvshuihe Forest Park (505 m)	OP013317
BBlni_7	*B. nigra* **sp. nov.**	male	CHINA: Yunnan, Gejiu, Lvshuihe Forest Park (505 m)	OP013318
BBlni_9	*B. nigra* **sp. nov.**	male	CHINA: Yunnan, Gejiu, Lvshuihe Forest Park (505 m)	OP013319
BBlni_10	*B. nigra* **sp. nov.**	male	CHINA: Yunnan, Gejiu, Lvshuihe Forest Park (505 m)	OP013320
BBlxj_1	*B. xinjiangica* **sp. nov.**	male	CHINA: Xinjiang, Dushanzi, Duku Highway (44°5′36″ N 84°44′59″ E, 1397 m)	OP013321
BBlxj_4	*B. xinjiangica* **sp. nov.**	male	CHINA: Xinjiang, Dushanzi, Duku Highway (44°5′36″ N 84°44′59″ E, 1397 m)	OP013322
BBlxj_6	*B. xinjiangica* **sp. nov.**	male	CHINA: Xinjiang, Dushanzi, Duku Highway (44°5′36″ N 84°44′59″ E, 1397 m)	OP013323
BBlxj_8	*B. xinjiangica* **sp. nov.**	female	CHINA: Xinjiang, Zhaosu, Qiongbola Forest Park (43°26′3″ N 81°1′13″ E, 1976 m)	OP013324
BBlxj_9	*B. xinjiangica* **sp. nov.**	female	CHINA: Xinjiang, Zhaosu, Qiongbola Forest Park (43°26′3″ N 81°1′13″ E, 1976 m)	OP013325

**Table 2:** Genetic distances between *Blepharicera* Macquart, 1843 COI sequences.

Species (Number of Sequences)	*Balangshana* (3)	*Beishanica* (6)	*Dushanzica* (4)	*Gengdica* (1)	*Kongsica* (5)	*Nigra* (9)	*Xinjiangica* (5)	*Fasciata* (3)
*balangshana* (3)	0 *							
*beishanica* (6)	18.3–18.5%	0–0.5% *						
*dushanzica* (4)	13.9–14.1%	15.7–16.6%	0.2–0.7% *					
*gengdica* (1)	16.2%	18.0–18.2%	13.1–13.7%	\				
*kongsica* (5)	15.2–15.4%	19.8–20.3%	13.7–14.3%	8.1–8.3%	0–0.2% *			
*nigra* (9)	15.4–16.2%	16.4–17.5%	16.4–17.5%	15.2–15.8%	14.5–15.6%	0–1.7% *		
*xinjiangica* (5)	13.9–14.5%	14.6–15.4%	5.4–6.2%	13.9–14.3%	14.5–15.4%	16.2–17.7%	0.2–1.2% *	
*fasciata* (3)	17.1–17.4%	17.4–17.8%	12.7–13.2%	15.8–16.0%	16.1–16.5%	14.5–15.1%	13.5–13.8%	0–0.3% *

* Intraspecific distances.

**Table 3:** Checklist of Chinese *Blepharicera* species with distribution data.

Species	Distribution	Chinese Region(s)	World Region(s)	Endemic to China
*B. asiatica* (Brodsky, 1930)	China (Yunnan, Guangxi); Afghanistan; India; Pakistan; Russia; Sri Lanka.	South China, Southwest China	Palaearctic, Oriental	
*B. balangshana* Zhang *et* Kang, 2022	China (Sichuan)	Southwest China	Palaearctic	+
*B. beishanica* **sp. nov.**	China (Qinghai)	Northwest China	Palaearctic	+
*B. dimorphops* Alexander, 1953	China (Fujian)	East China	Oriental	+
*B. dushanzica* **sp. nov.**	China (Xinjiang)	Northwest China	Palaearctic	+
*B. gengdica* Zhang *et* Kang, 2022	China (Sichuan)	Southwest China	Palaearctic	+
*B. hainana* Kang *et* Yang, 2014	China (Hainan)	South China	Oriental	+
*B. hebeiensis* Kang *et* Yang, 2014	China (Hebei, Shanxi)	North China	Palaearctic	+
*B. kongsica* Zhang *et* Kang, 2022	China (Sichuan)	Southwest China	Palaearctic	+
*B. macropyga* Zwick, 1990	China (Hainan)	South China	Oriental	+
*B. nigra* **sp. nov.**	China (Yunnan)	Southwest China	Oriental	+
*B. taiwanica* (Kitakami, 1937)	China (Taiwan)	East China	Oriental	+
*B. uenoi* (Kitakami, 1937)	China (Taiwan)	East China	Oriental	+
*B. xinjiangica* **sp. nov.**	China (Xinjiang)	Northwest China	Palaearctic	+
*B. xizangica* Kang, Zhang *et* Yang, 2022	China (Xizang)	Southwest China	Palaearctic	+
*B. yamasakii* (Kitakami, 1950)	China (Heilongjiang)	Northeast China	Palaearctic	+

## Data Availability

The mitochondrial COI sequence data sequenced in this study are openly available in GenBank of NCBI [33] under the accession nos. OP013293–OP013325.

## Supplementary Materials

The following supporting information can be downloaded at: www.mdpi.com/article/10.3390/insects13090794/s1, Table S1: Details of genetic distances between *Blepharicera* Macquart, 1843 COI sequences.
